# Extensive Degenerative Change Masquerading Histomorphology in a Giant Cystic Gastrointestinal Stromal Tumor With Rare PDGFRA Mutation

**DOI:** 10.7759/cureus.10772

**Published:** 2020-10-02

**Authors:** Jawaria Rahman, Syed Rahmanuddin, Sunder Sham, Snehal Sonawane

**Affiliations:** 1 Department of Pathology, Case Western Reserve University School of Medicine, Cleveland, USA; 2 Department of Radiology, City of Hope Comprehensive Cancer Center, Duarte, USA; 3 Department of Pathology, Lenox Hill Hospital Northwell Health, New York, USA; 4 Department of Pathology, South Bend Medical Foundation, South Bend, USA

**Keywords:** mutations, gastrointestinal stromal tumor, gist, pdgfra, dog-1

## Abstract

Gastrointestinal stromal tumors (GISTs) are the most frequent type of mesenchymal tumors of the gastrointestinal (GI) tract, and most of the time they acquire the mutation of special kinds of genes. GISTs may be familial or inherited and affect several family members of the family or can be sporadic. The risk of GIST is increased in people with mutations in the receptor tyrosine kinase (KIT) and platelet-derived growth factor receptor alpha (PDGFRA) genes. In this report, we present a case of a large GIST with extensive cystic and degenerative change in a 76-year-old female patient with a rare Asp842-His845 deletion mutation detected in PDGFRA exon 18, that required subtotal gastrectomy with en bloc resection.

## Introduction

Gastrointestinal stromal tumors (GISTs) are malignant tumors arising from special kinds of cells in the wall of the gastrointestinal (GI) tract called the interstitial cells of Cajal (ICCs) [1]. More than half of GISTs, around 50%-70%, start in the stomach, while 20%-45% develop in the small intestine [2]. Their prognosis is substantially changeable, as are their mitotic rate, site, and size [3]. Receptor tyrosine kinase (KIT) and platelet-derived growth factor receptor alpha (PDGFRA) gene mutations are deemed to be associated with GIST pathogenesis. No mutations are found in 5-10% of tumors, and those variants are called wild type (WT) GIST [4]. Histopathologically, the diagnosis of a GIST depends on immunohistochemistry and morphology [5]. Surgery is the primary treatment of GISTs. However, recurrence is observed in approximately 40% of patients after the resection [2]. The object of the study was to analyze GISTs to obtain a more comprehensive picture of their natural history, genetic attributes, relative frequency, and clinicopathologic and immunohistochemical features; particularly, establishing the knowledge of a rare novel molecular mutation related to the PDGFRA gene.

## Case presentation

A 76-year-old woman admitted to the hospital with abdominal pain and bloating. She was unable to tolerate any oral intake for several days and also complained of a decrease in appetite, nausea, vomiting, constipation, and weight loss of 20 lb over three months. She had a past surgical history of open cholecystectomy with appendectomy and total abdominal hysterectomy. A review of systems was normal, except the respiratory system, which was positive for shortness of breath but negative for cough. No chest pain and palpitations were present.

On physical examination, the patient was awake and alert with no acute distress. Pupils were equal, round, and reactive to light and accommodation. She had a regular rate and rhythm - no increased work of breathing. The abdomen was grossly distended, firm, and nontender; a well-healed scar from open cholecystectomy was present on the right side of the abdomen. Extremities were warm with regular pulses. The laboratory evaluation showed anemia with hemoglobin (HGB) of 8.1g/dl and hematocrit (HCT) of 24.7g/dl. Other readings included white blood cell (WBC) 4.1, platelets 289, cancer antigen (CA)-125 7, carcinoembryonic antigen (CAE) 1.8, and CA-19-9 24, all within normal limits.

Imaging findings

Computed Tomography (CT) scan of the abdomen/pelvis revealed a mass of 30 x 27 x 20 cm predominantly massive, cystic appearing lesion occupying much of the abdomen and pelvis, resulting in a significant mass effect upon all surrounding structures. Low-grade obstruction related to mass effect was also noted. Imaging studies were unable to determine the site and side of the origin. Given her symptoms and radiological findings, the recommendation was made to resect this mass.

Intraoperative findings showed a large intraabdominal cystic mass measuring at least 30 cm. The cystic mass was incised, and the fluid contents were suctioned out, with at least 4 liters of fluid being removed. The components of the cyst appeared to be hemorrhagic and necrotic. The mass was inseparable from the posterior portion of the stomach. Therefore, subtotal gastrectomy was performed with en bloc resection of the mass. Moreover, transverse colectomy was also carried out due to the dense adherence of the cyst to the colon. Part of the cyst wall was submitted for the frozen section, which showed necrotic and degenerated tissue fragments.

Pathologic findings

**Figure 1:**
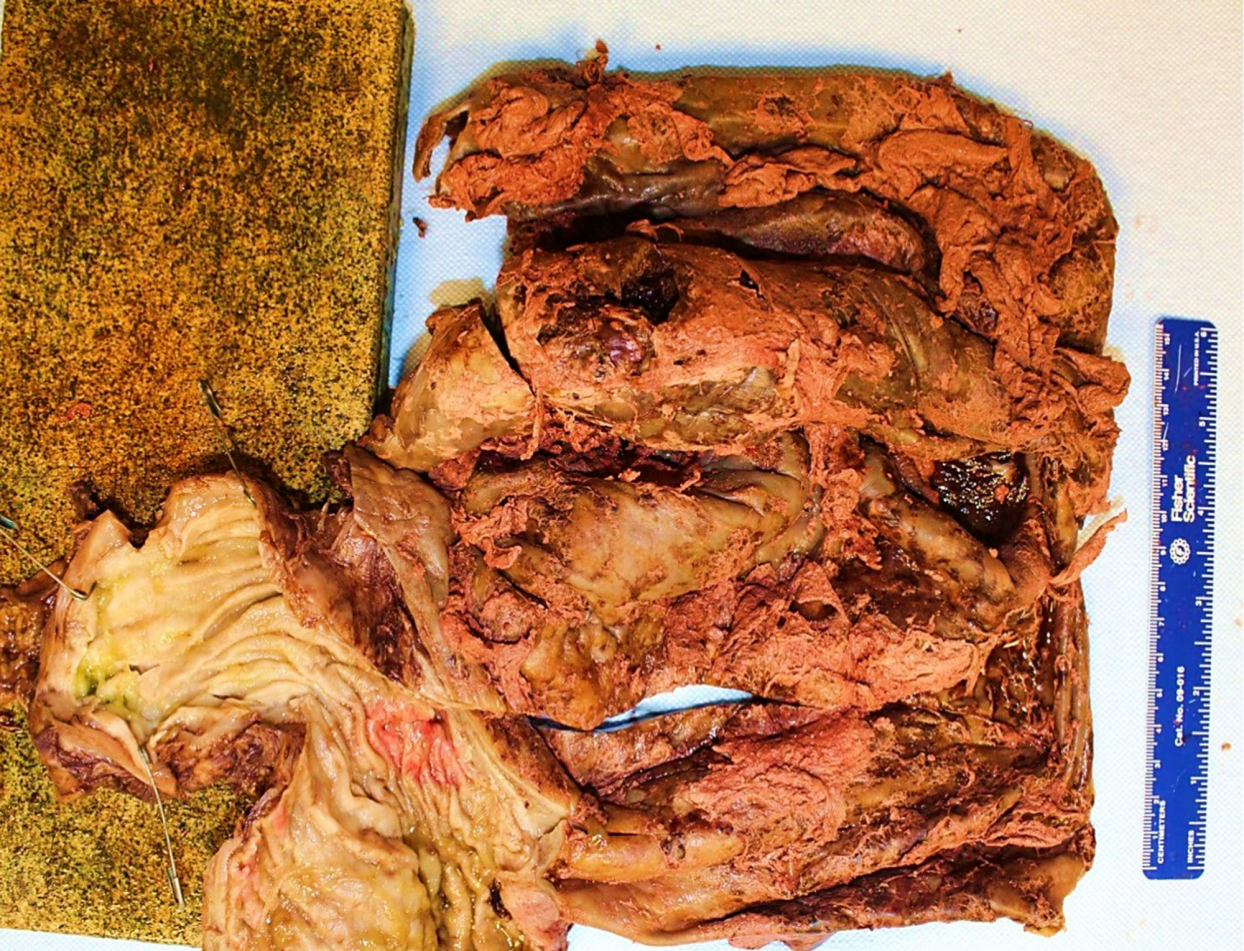
The macroscopic examination of the specimen showed the variegated appearance of the cyst wall with hemorrhage and necrosis.

**Figure 2:**
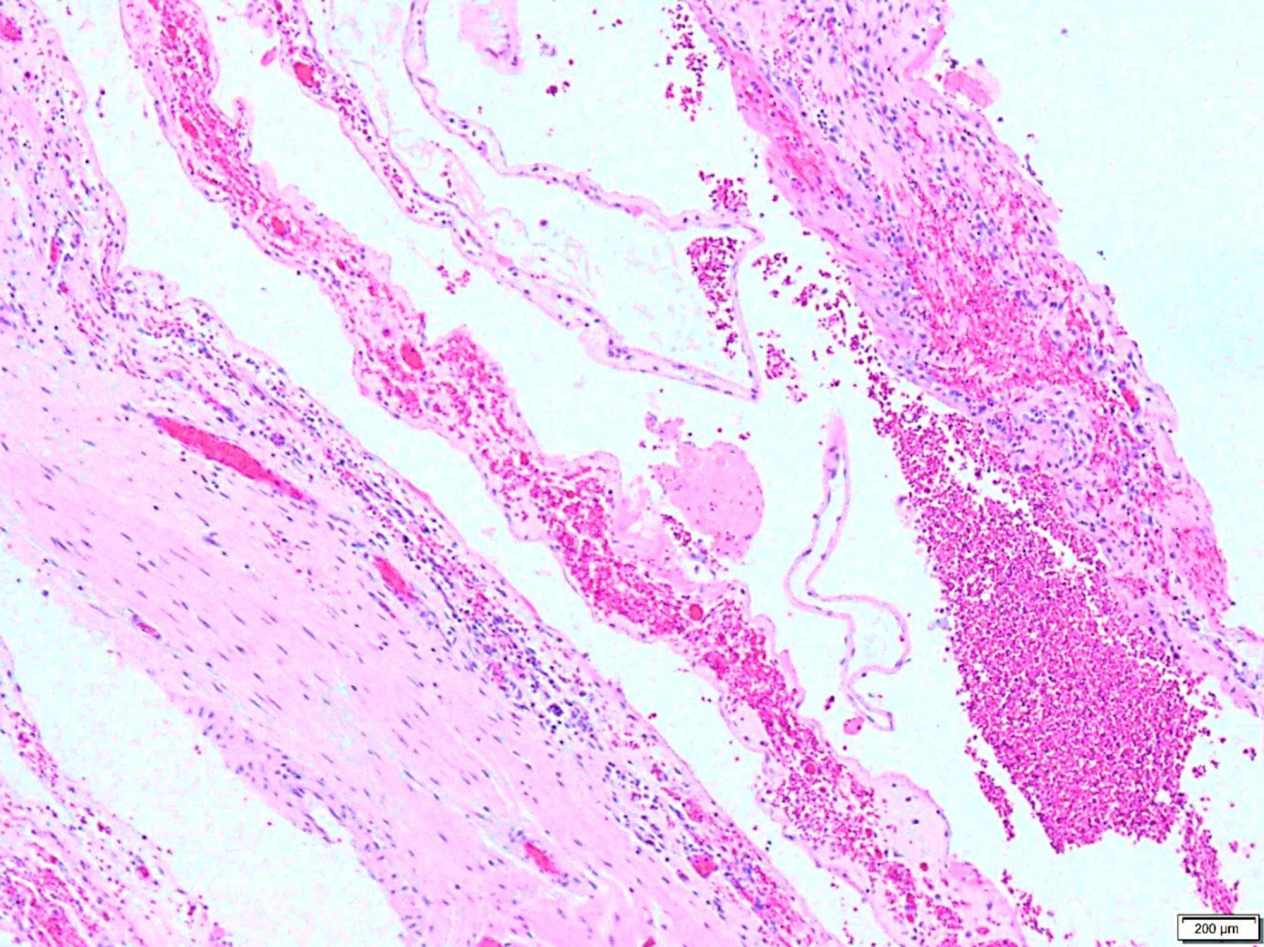
A low power view of the predominantly hemorrhagic appearance of the cyst wall.

**Figure 3:**
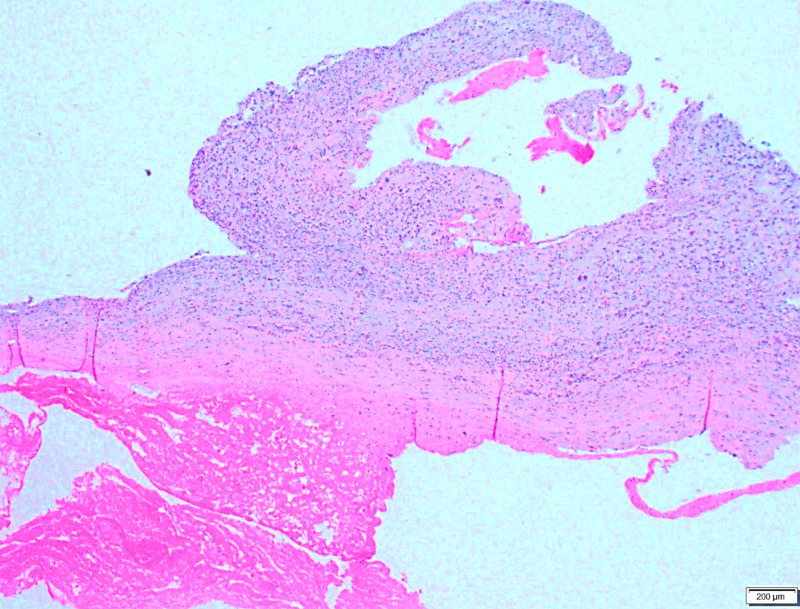
A low power image of the cyst wall with area and hemorrhage and viable tumor cells attached to the cyst wall.

**Figure 4:**
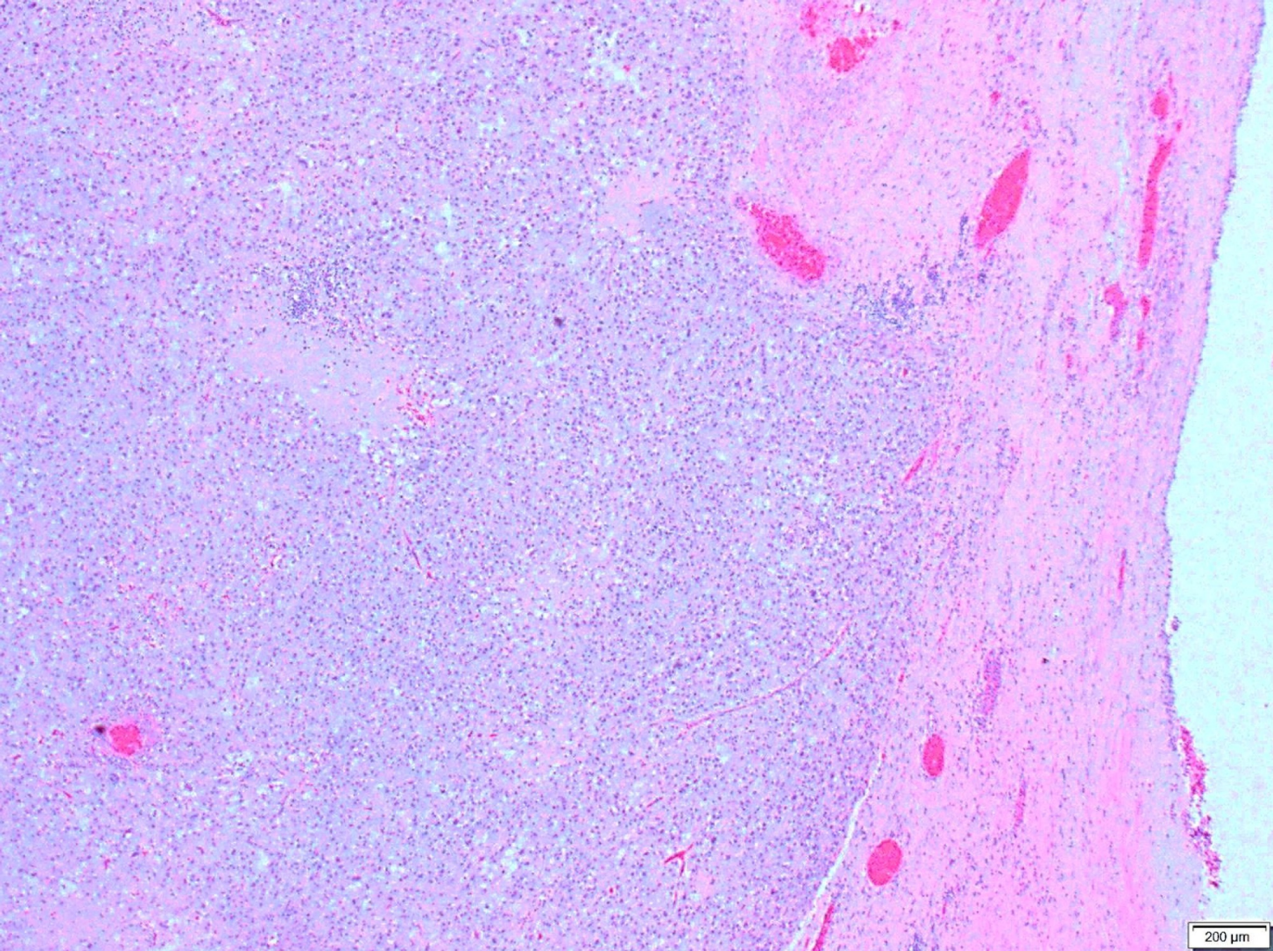
A medium power image of the cyst wall area focusing the viable tumor cells arranged in sheets and have pink to clear cytoplasm.

**Figure 5:**
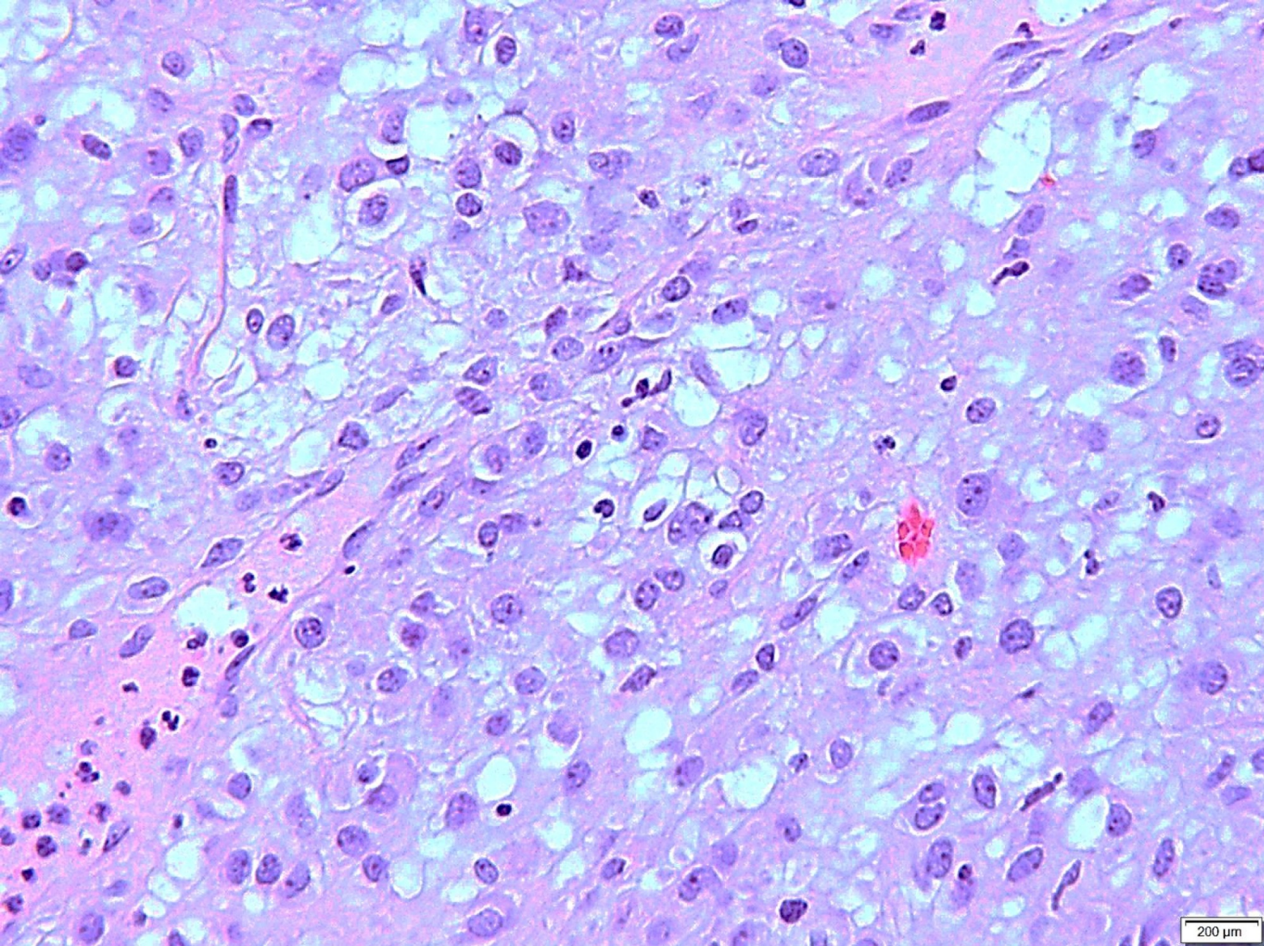
A high power view of the tumor with a round to polygonal cells, pale eosinophilic to clear cytoplasm and round nuclei.

**Figure 6:**
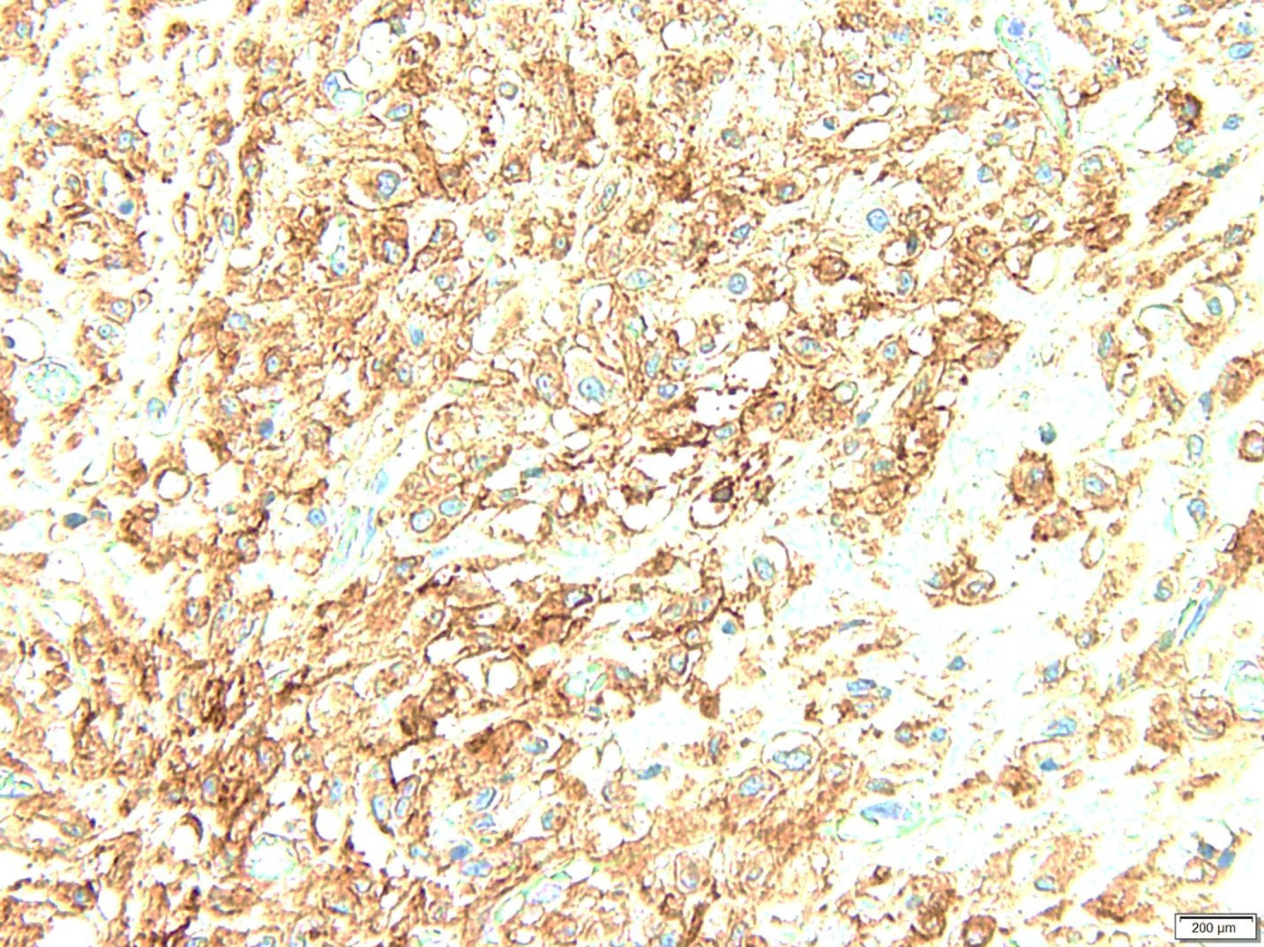
DOG-1 immunohistochemical stain positive in tumor cells. DOG-1: Discovered on GIST-1

On macroscopic examination, the cystic mass measured 33.3 cm in the greatest dimension with extensive intramural hemorrhage and degeneration (Figure 1). Histopathologic examination of multiple sections from the cyst wall showed an extensively necrotic cyst wall with hemorrhage (Figure 2). Very few sections showed cyst wall lined by round to oval cells with very pale to clear cytoplasm with benign-appearing nuclei. Few nuclei demonstrated prominent nucleoli (Figures 3, 4, 5). Rare mitosis was identified. The differential diagnosis included pecoma, liposarcoma, paraganglioma, GIST, mesothelioma, ovarian theca cell tumor, melanoma, renal cell carcinoma, and epithelioid leiomyoma. The immunohistochemical stains for keratins (AE1/AE3, CK8/18), CD117, human melanoma black 45 (HMB45), S100, and activin receptor-like kinase 1 (ALK1) were performed. However, the lesional cells were only positive for Discovered on GIST-1 (DOG-1) (Figure 6).

Based on the above findings, the diagnosis of GIST with epithelioid morphology was favored, and tissue was sent for KIT and PDGFRA mutational analysis. Pieces of evidence based on the sequence analysis, Asp842-His845 deletion was detected on the PDGFRA gene exon 18, which confirmed the diagnosis of PDGFRA-mutated GIST, predominantly epithelioid type with 90% necrosis. It was histologically low grade and had a pathological stage of pT4pN0 and was categorized as moderate risk.

The patient is doing well after surgery. It supports the fact that complete surgical resection improves local recurrence rate and overall survival.

## Discussion

Gastrointestinal stromal tumors (GISTs) are specific KIT- or PDGFRA-signaling driven tumors, which were initially classified as smooth muscle tumors, and now understood to be a separate, distinct group of mesenchymal tumors specific to the GI tract. A majority of these tumors (60%-70%) present in the stomach [6-8]. Others, approximately 20%-45%, develop in the small intestine; however they occur throughout the GI tract [2-4]. A very few also arise outside the GI tract, such as the mesentery, peritoneum, and omentum. GISTs may occur at any age; however, they are most frequently diagnosed in patients above 50 years of age [7]. The aggressiveness, location, and the size of the tumor determines the severity of the disease. It may present with bloody or tarry stools, hematemesis, and anemia caused by chronic bleeding. Other symptoms include tender and swollen abdomen, inability to take orally, and GI obstruction [9,10], as seen in our case.

Genetic attributes

The risk of GIST is increased in people with mutations in the KIT and PDGFRA genes [10,11]. The most frequent KIT mutations are identified in exons 13, 14, 17 (1% each), exon 9 (10-13%), and exon 11 (66-71%). The PDGFRA mutations (8%) are detected in exon 12 (1%), 14 (1%), and 18 (5-6%). Besides, 5-10% of tumors are identified as without mutations, and those subtypes are called wild type (WT) GIST [4]. Imatinib is a kinase inhibitor medication utilized to treat metastatic and unresectable gastrointestinal stromal tumors. In light of the type of GIST diagnosed in our case, the patient did not receive imatinib therapy. Imatinib acts by blocking the action of the abnormal protein that signals malignant cells to increase [12]. The PDGFRA-mutant GISTs are highly resistant to imatinib, and total imatinib resistance is more frequent in PDGFRA-mutant and wild type tumors than in KIT-mutant tumors [13].

There is an enormous amount of literature explaining the pathogenesis of GISTs associated with PDGFRA mutations, which is one of the pathways seen in 3-7% of GIST cases. Further analysis of PDGFRA mutations shows that the most common variation is located on exon 18 and p, of which D842V was most frequent (around 65%). In our case report, the GIST mutation was a deletion on exon 18 in PDGFRA, which involved the deletion of Asp842-His845 codons. This is a rare mutation of about 1% [14]. A gene mutation analysis performed by Li et al. on 827 patients revealed only two cases of deletion of Asp842-His845 codons [15]. Kanda et al. also described the same mutation deletion of Asp842-His845 codons in a nine cm GIST in the stomach of a 44-year-old man, which led to a rupture and caused hemoperitoneum. The histopathologic examination was consistent with epithelioid GIST, and despite rupture, the patient did well postoperatively [16]. Dileo et al. reported one case of this mutation and found that these cases showed a partial response to treatment with imatinib [17]. A study conducted by Corless et al. supported the fact that deletion mutations of 842-845 of exon 18 in PDGFRA were sensitive to imatinib [18]. In our case, the tumor did not rupture and was excised completely, so imatinib therapy was not offered, and the patient is doing well.

Gross and histopathologic features

A study by Miettinen et al. demonstrated the gross features of GIST, which varied widely. In most cases, the tumors were described as large, well-demarcated, nodular or lobulated, with cystic degeneration, which is not uncommon, and was seen in our case. Moreover, the study showed the cut surface of the tumors, which demonstrated various morphology, and the most common description was tan-pink, yellowish to gray-white appearance often mottled by hemorrhagic discoloration. The consistency of the tumor varied but was typically firm in small and benign tumors, soft fish-fleshy or gelatinous in malignant tumors, and commonly porous-hemorrhagic in larger tumors. Our case showed an extensive cystic tumor with variegated appearance, intratumoral hemorrhage, necrosis, and cystic degeneration. Other recorded features include necrosis, ulceration, calcification, hyalinization, and organoid pattern. Further, the study described other histologic variants include sclerosing, palisaded-vacuolated, hypercellular, sarcomatous with significant nuclear atypia and high mitotic activity, which were specific for spindle cell tumors and occurred in 70% of cases, whereas epithelioid tumors, found in 20% of cases, showed sclerosing, dyscohesive, hypercellular, sarcomatous with substantial nuclear atypia and low mitotic activity [6].

**Figure 7:**
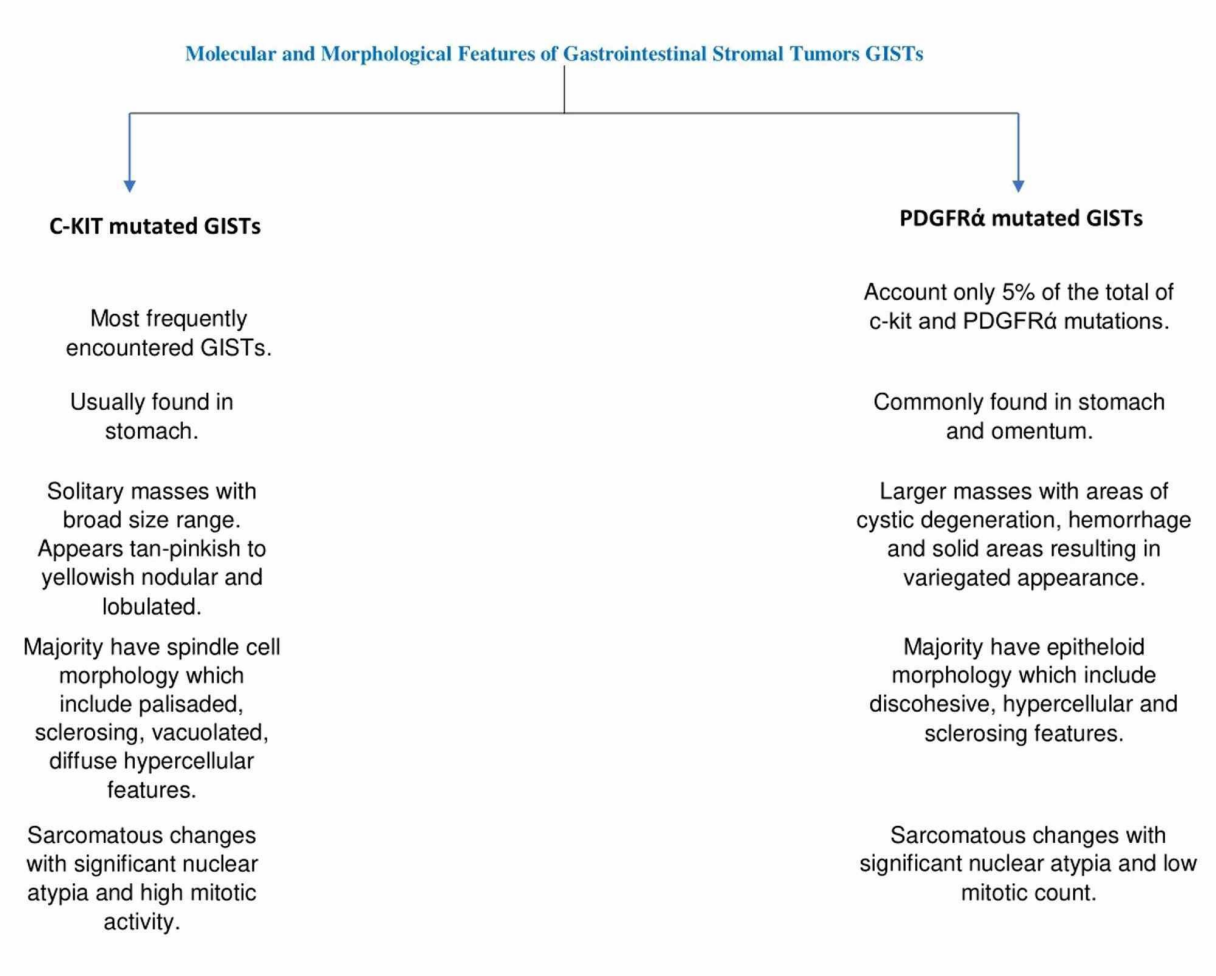
Molecular and morphological features of Gastrointestinal Stromal Tumors (GISTs)

Our case demonstrated a very distinct epithelioid morphology consisting of relatively discohesive sheets of round to oval cells with very pale eosinophilic to clear cytoplasm and relatively benign-appearing nuclei with rare cells displaying prominent nucleoli. This morphologic appearance can be attributed to secondary changes such as intertumoral hemorrhage and cystic degenerative change. The outcome has significantly relied on tumor size and mitotic activity. Nuclear atypia was arbitrarily defined by nuclear enlargement, increased nucleocytoplasmic ratio, hyperchromasia, vesicular nature of the chromatin, or pleomorphism. Based on the extent, it was classified as focal or diffuse, and based on estimated severity, called as mild or moderate. Morphological features of subtypes of GISTs are shown in Figure 7 [6-8].

Immunohistochemical features

A study conducted by Tepeoglu et al. showed immunohistochemically stained GISTs with smooth muscle actin (SMA), CD117, desmin, CD34, and S100. Furthermore, Discovered on GIST-1 (DOG1), a novel gene on gastrointestinal stromal tumors that encodes chloride channel protein was utilized in CD117-negative cases, as we have confirmed in our case. The diffuse pattern was seen in the staining of CD117 if the number of positive cells was ≥50% and focal if <50%. Immunohistochemically, 90.8% of GIST cases are CD117 positive, whereas CD34+ (73.3%), smooth muscle actin+ (61.7%), desmin+ (11.7%) and S100+ (28.3%). All CD117-negative cases express DOG-1 [19]. Our case was only DOG-1 positive and helped to clinch the diagnosis.

## Conclusions

It is imperative to keep the differential diagnosis of cystic GIST in mind when we come across large cystic masses in the abdomen. Ongoing advances in molecular diagnostic and genomic studies have empowered us to recognize new changes that could be causally connected with GIST advancement. Besides, extensive studies, including large clinical trials, are continuing to build a promising role in the mutation testing techniques for better survival in GIST patients. This case also emphasizes the significance of a thorough examination of the tissue and utilization of appropriate immunohistochemical markers (e.g., DOG-1 in CD117-negative GIST). However, surgical resection remains the gold standard treatment for GIST. The availability of imatinib mesylate, a KIT and PDGFRA tyrosine kinase inhibitor, has built its significance to diagnose these cases. Besides, this tyrosine kinase inhibitor has also made it important to understand the natural history of these tumors and optimize the treatment properly.
